# Analysis of influencing factors of shear wave elastography of the superficial tissue: A phantom study

**DOI:** 10.3389/fmed.2022.943844

**Published:** 2022-08-08

**Authors:** Qiyang Chen, Baixue Shi, Yang Zheng, Xiangdong Hu

**Affiliations:** ^1^Department of Ultrasound, Beijing Friendship Hospital, Capital Medical University, Beijing, China; ^2^AML, Department of Engineering Mechanics, Institute of Biomechanics and Medical Engineering, Tsinghua University, Beijing, China

**Keywords:** ultrasound, shear wave elastography (SWE), phantom, superficial, influencing factors

## Abstract

Shear wave elastography (SWE) is widely used in clinical work. But there is no standard protocol and operation specification for SWE acquisition methods, which impacts the diagnosis and clinical staging. This study aimed to investigate the influence factors of diameter, depth, and stiffness on SWE using different probes at superficial depths and discuss SWE differences with two machines at superficial depths. We performed SWE on two elastic phantoms that each phantom contained six subjects with two stiffness (41.06 ± 4.62 kpa and 57.30 ± 4.31 kpa), three diameters (10, 15, and 18 mm), and two depths (15 and 25 mm). A total of 240 measurements were obtained by using two ultrasound machines (SuperSonic Imagine Aixplorer and Mindray Resona 7) and 4 probes (SL15-4 and SL10-2, L11-3, and L14-5). The measurements were compared among 4 probes, 3 diameters, and 2 depths. There was no significant difference in SWE measurements among the probes from the same machine. The SWE measurements were affected by diameter, and the degree of influence was related to the stiffness. The SWE measurements were unaffected at a 15–25 mm depth range.

## Introduction

Shear wave elastography (SWE) is a novel technique and is widely used in clinical practice due to its non-invasive procedure and visualization property (1). The principle of SWE is to utilize acoustic radiation force impulse (ARFI) to produce shear waves for imaging, then the shear wave conduction velocity (SWV) is measured and converted into Young’s modulus of tissue (2–4). While measuring SWV of hard lesions, a larger Young’s modulus is obtained as shear waves travel faster in a hard lesion (5). It is generally acknowledged that the stiffness of malignant tumors is always harder than benign lesions (6). Therefore, SWE can be used for the differential diagnosis of benign and malignant lesions by obtaining the elasticity of the tissue (7, 8).

In recent years, the application of SWE in the breast, thyroid, and other superficial organs has gradually increased (9–11). Clinicians can quantitatively obtain the stiffness of lesions through SWE. However, we found that many studies have shown great differences in the cut-off value. In thyroid lesions, some studies (12) concluded that the cut-off value for thyroid nodules was 27.49 kpa which was lower than the optimal cut-off value of 67.3 kpa obtained by Baig et al. (13) and 85.2 kpa by Park et al. (14). Some scholars believe that this is due to a certain difference in the thyroid tissue structure between Chinese and Western populations (15). In breast lesions, a previous study (16) showed that the cut-off value of benign and malignant breast nodules in the Chinese population was 24.7 kpa, which was much lower than the optimal cut-off value of 80 kpa obtained by Min et al. (17). Since some previous studies have shown that higher SWE measurement values were closely related to poor prognosis, the presence of these conditions has an impact on the diagnosis and clinical staging (18, 19). We think this is due to the lack of protocol and operation specifications for SWE acquisition methods in clinical work, which hinders the further development of this technology. Previous studies (20–22) have reported that different manufacturer systems, probes, and acquisition depths can affect the SWV and Young’s modulus. Therefore, we believe that it is necessary to explore the influencing factors of SWE. However, as far as we know, there are few relevant studies, and the types of manufacturer systems and probes discussed are not extensive. Therefore, a study covering more imaging systems, probes, and other influence factors will help to formulate a more perfect SWE measurement standard.

The purpose of this study was to investigate the influence factors of diameter, depth, and stiffness on SWE using different probes from different machines at superficial depths.

## Materials and methods

### Phantom preparation

Two custom-made elastic tissue phantoms (Phantom 1 and 2) were fabricated by gelatin with tumor-mimicking subjects which were provided by the Department of Engineering Mechanics, the Institute of Biomechanics and Medical Engineering, Tsinghua University. The production process was consistent with that in the study by Zhang et al. (23).

**FIGURE 1:**
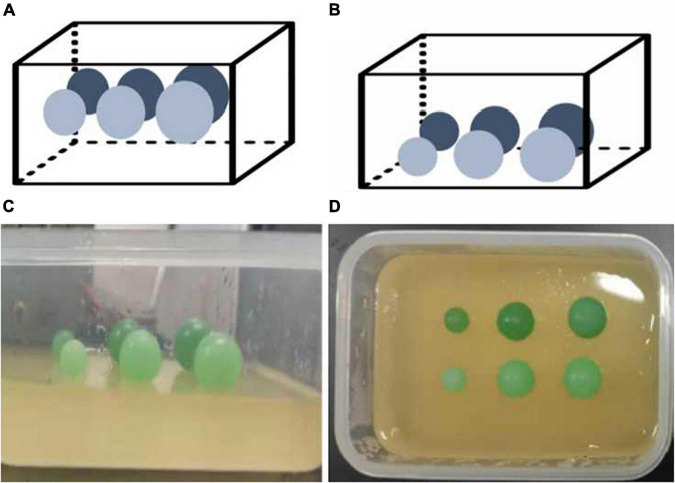
Elastic tissue-mimicking phantoms. The schematic and pictures show the phantoms with subjects within the different sizes, depths, and stiffnesses in the matrix. **(A)** Phantom 1; **(B)** Phantom 2; **(C,D)** gelatin phantoms.

As shown in Figure 1, each phantom consisted of a soft matrix of gelatin and six subjects located at the same depth of 1.5 cm for Phantom 1 and 2.5 cm for Phantom 2. Each row had three subjects with diameters of 10, 15, and 18 mm in size with respective stiffness values of 41.06 ± 4.62 kpa (stiffness 1) and 57.30 ± 4.31 kpa (stiffness 2).

### Machines and methods

Two ultrasound machines were used in this study including Aixplorer (SuperSonic Imagine, Aix-en-Provence, France) with transducers of SL15-4 and SL10-2 and Resona 7 (Mindray, Shenzhen, China) with transducers of L11-3 and L14-5. Both systems were equipped with an SWE mode. The Young’s modulus can be calculated according to the equation: *E* = 2 × (1 + ν) × ρ × (SWV)^2^, the E is Young’s modulus, ν is the Poisson ratio (0.5), and ρ is the density (estimated as 1.0 g/cm^3^) (24).

Routine thyroidal settings were applied while taking the SWE measurements. The maximal cross-section of the phantom subjects was observed by the B-mode imaging. Then, the instrument was switched to the SWE mode and the sampling frame was placed in the center of the image within the subject.

The SWE imaging was frozen and measurements of the stiffness were performed after 5 s of real-time imaging for stabilization. The region of interest (ROI) of SWE was placed in the center of the phantom subject and the diameter of the ROI was adjusted according to the size of the target. The mean Young’s modulus E (E_mean_) in the ROI was measured and repeated 5 times. The mean value of each subject (E_i_) was calculated by averaging these values. In this experiment, each subject (*n* = 12) was measured by each of the probes (*n* = 4) in both systems. Thus, a total of 240 E_mean_ values and 48 E_i_ values were obtained.

The phantoms were stored in a refrigerator at 8°C before measurement. The SWE measurements were tested at room temperature within half an hour.

#### Statistical analysis

Statistical analyses were performed using IBM SPSS Statistics Version 26.0 (IBM, Armonk, NY, United States). All the data obtained from the experiments passed the normality tests. The Friedman test was used to compare the E_i_ values among the four probes and three diameters. If the data followed a normal distribution, the paired-samples *t*-test was used to compare the E_i_ values between two depths; otherwise, the Wilcoxon signed-rank test was used. Bland–Altman plot tested the difference between two machines. A *p*-value less than 0.05 was considered statistically significant.

The coefficient of variation (CV) was used to evaluate the precision and repeatability of the measurements The smaller the CV, the better the repeatability of the measurement. The percentage error was calculated as follows: | actual stiffness values – E_i_| /actual stiffness values × 100%. When the percentage error was close to zero, the measured value could accurately reflect the actual stiffness values of phantom subjects.

## Results

The results of the SWE measurement value in two ultrasound machines with four probes are summarized in Table 1.

**TABLE 1:** The shear wave elastography (SWE) measurement values, coefficient of variation, and percentage error obtained using four probes.

Probe	Diameter (mm)	Depth (mm)	Stiffness 1 (41.06 ± 4.62 kpa)			Stiffness 2 (57.30 ± 4.31 kpa)		
			E_i_ (kpa)	CV^a^ (%)	Percentage error^1^ (%)	E_i_ (kpa)	CV^a^ (%)	Percentage error^2^ (%)
L11-3	10	15	29.51 ± 0.86	2.92	28.14	41.38 ± 0.56	1.35	27.78
		25	32.91 ± 0.54	1.63	19.86	39.26 ± 0.70	1.77	31.49
	15	15	33.21 ± 0.24	0.73	19.13	42.73 ± 0.56	1.30	25.43
		25	32.37 ± 0.44	1.36	21.17	41.70 ± 0.78	1.86	27.22
	18	15	30.05 ± 0.17	0.57	26.82	40.82 ± 0.37	0.91	28.76
		25	30.44 ± 0.42	1.37	25.85	43.29 ± 0.06	0.14	24.45
L14-5	10	15	30.20 ± 0.17	0.52	19.15	39.17 ± 0.45	1.14	31.63
		25	32.31 ± 0.56	1.72	21.31	40.28 ± 0.76	1.90	29.70
	15	15	30.44 ± 0.42	0.3	27.89	40.92 ± 0.27	0.65	28.59
		25	33.54 ± 0.46	1.39	18.30	41.38 ± 0.34	0.81	27.78
	18	15	35.01 ± 0.28	0.79	14.72	38.16 ± 1.37	3.59	33.41
		25	33.68 ± 0.30	0.9	17.98	34.32 ± 0.20	0.58	40.11
SL15-4	10	15	36.04 ± 0.49	1.35	12.23	44.62 ± 3.20	7.16	22.13
		25	38.78 ± 0.49	1.26	5.55	47.60 ± 1.45	3.06	16.93
	15	15	40.76 ± 0.25	0.61	0.73	52.44 ± 0.80	1.53	8.48
		25	40.08 ± 0.50	1.25	2.39	55.12 ± 1.15	2.08	3.80
	18	15	36.66 ± 0.36	0.99	10.72	49.00 ± 1.07	2.19	14.49
		25	37.66 ± 0.10	0.27	8.28	49.54 ± 1.21	2.43	13.54
SL10-2	10	15	30.84 ± 0.87	2.82	24.89	46.38 ± 1.22	2.64	19.06
		25	32.96 ± 0.98	2.96	19.73	42.96 ± 0.57	1.34	25.03
	15	15	35.76 ± 0.81	2.27	12.91	50.04 ± 1.43	2.85	12.67
		25	33.44 ± 0.44	1.32	18.56	47.86 ± 1.01	2.11	16.47
	18	15	31.60 ± 0.18	0.57	23.04	44.96 ± 1.22	2.72	21.54
		25	30.74 ± 0.24	0.79	25.13	42.74 ± 1.29	3.02	25.41

^a^ Coefficient of variation.

^1^ Represents that the stiffness of the phantom is 41.06 ± 4.62 kpa.

^2^ Represents that the stiffness of the phantom is 57.30 ± 4.31 kpa.

### Comparison of the agreement between machines

**FIGURE 2:**
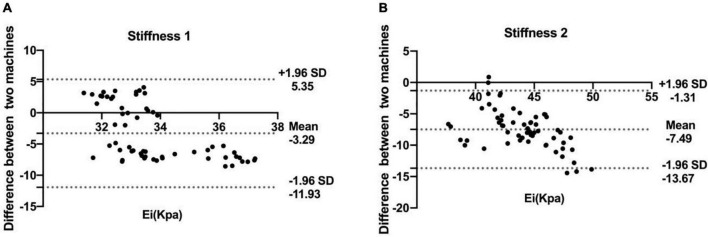
Bland–Altman plots of the shear wave elastography (SWE) measurement values obtained by the two machines at stiffness 1 **(A)** and stiffness 2 **(B)**. The solid lines represent the mean difference in the SWE measurement values between two machines; the dashed lines define the LoA (mean of the differences ± 1.96 SD).

The Bland–Altman plots show the agreement between two machines. Figure 2 shows that the mean difference between the two machines was −3.29 and −7.49 kPa for the phantoms of stiffness 1 and stiffness 2, respectively. The limits of agreement (LoA) values ranged from −11.93 to 5.35 and −13.67 to −1.31 for the phantoms of stiffness 1 and stiffness 2, respectively. The LoA values of stiffness 2 were larger than stiffness 1.

### Comparison of shear wave elastography measurements among probes

**FIGURE 3:**
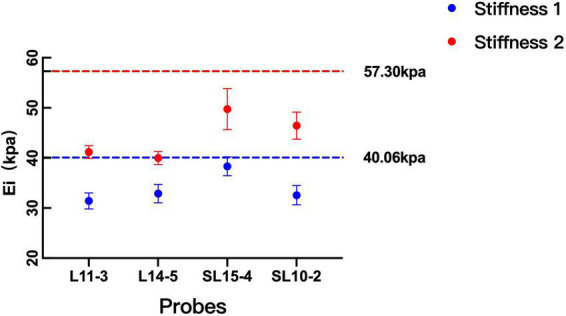
Comparison between the measurement values among probes. The measurements on both probes were lower than the theoretical values of the two kinds of stiffness. With the increase in the stiffness of the phantom, the difference between machines becomes significant.

Figure 3 depicts the difference in SWE measurement values between the four probes without considering other influencing factors. For stiffness 1, the difference between SL15-4 and other probes (L11-3, L14-5, and SL10-20) was statistically significant (*p* = 0.000). For stiffness 2, the difference between probes from different machines is statistically significant (*p* = 0.000), on the contrary, the difference between probes of the same machine is not statistically significant (*p* > 0.05).

The comparison of SWE measurement values of four probes for phantom subjects with different diameters, stiffness, and depth are summarized in Table 2. In general, the difference between L11-3 and L14-5 was statistically significant only when the phantom subjects at 15 mm depth with 18 mm diameter and stiffness 1 (*p* = 0.045), and the difference between SL15-4 and SL10-2 was statistically significant when the phantom subjects with 18 mm diameter and stiffness 1 (*p* = 0.045, *p* = 0.014) or at 15 mm depth with 10 mm diameter and stiffness 1 (*p* = 0.023). In other cases, the difference between the two probes under the same ultrasound machine was not statistically significant (*p* > 0.05).

**TABLE 2:** Comparison of measurement values of different phantom subjects between four probes.

Depth (mm)	Stiffness 1 (41.06 ± 4.62 kpa)						Stiffness 2 (57.30 ± 4.31 kpa)					
Diameters (mm)	15			25			15			25		
Probes	10	15	18	10	15	18	10	15	18	10	15	18
L11-3 VS L14-5	0.084	1.000	0.045*	1.000	0.346	0.129	0.466	1.000	1.000	1.000	1.000	0.269
L11-3 VS SL15-4	0.001*	0.045*	0.000*	0.084	0.001*	0.002*	1.000	0.062	0.045*	0.002*	0.010*	0.269
L11-3 VS SL10-2	1.000	1.000	1.000	1.000	0.617	1.000	0.466	0.893	1.000	0.129	0.415	1.000
L14-5 VS SL15-4	1.000	0.000*	1.000	0.003*	0.306	1.000	0.038*	0.001*	0.000*	0.014*	0.003*	0.000*
L14-5 VS SL10-2	0.727	0.045*	1.000	0.988	1.000	0.522	0.003*	0.033*	0.045*	0.523	0.170	0.269
SL15-4 VS SL10-2	0.023*	1.000	0.045*	0.223	0.159	0.014*	1.000	1.000	1.000	1.000	1.000	0.269

*Represents a statistical difference. VS represents a comparison between transducers using the Wilcoxon rank-sum test.

### Comparison of shear wave elastography measurements among diameters

**FIGURE 4:**
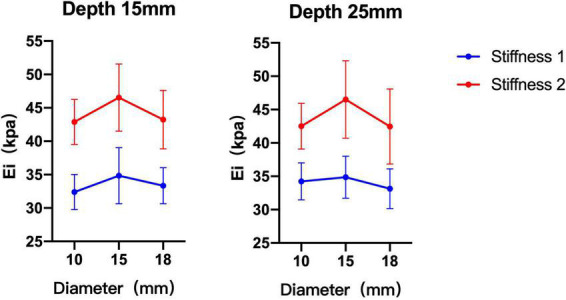
Comparison of shear wave elastography (SWE) measurement values of three diameter phantom subjects under different stiffness and depth. The measurements of 15 mm diameter phantom subjects were greater than 10 and 18 mm diameter phantom subjects.

The phantom subjects with a 15 mm diameter were closest to the theoretical values compared with 10 and 18 mm phantoms. Without considering the condition of probes, the difference between 10 and 15 mm phantoms was statistically significant when phantom subjects with stiffness 2 (depth 15 mm *p* = 0.011, depth 25 mm *p* = 0.020) The difference between the measurement values of 15 and 18 mm phantom was statistically significant when the phantom subjects with stiffness 1 at depth 25 mm or with stiffness 2 at depth 15 mm (*p* = 0.049, *p* = 0.017). There was no significant difference in the measurement values of 10 and 18 mm phantoms in all conditions (*p* > 0.05) (Table 3 and Figure 4).

**TABLE 3:** Comparison of measurement values of three diameters.

Depth (mm)	Stiffness 1 (41.06 ± 4.62 kpa)		Stiffness 2 (57.30 ± 4.31 kpa)	
Diameters (mm)	15	25	15	25
10 VS 15	0.108	0.242	0.011*	0.020*
15 VS 18	0.461	0.049*	0.017*	0.231
10 VS 18	0.314	0.114	0.445	0.445

*Represents a statistical difference. VS represents a comparison between diameters using the Wilcoxon rank-sum test.

### Comparison of shear wave elastography measurements among depths

**FIGURE 5:**
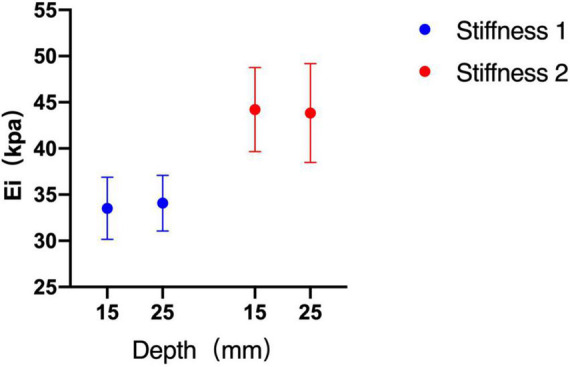
Shear wave elastography (SWE) measurements between depths of 15 and 25 mm. There was no significant difference between the two depths.

Without considering the influence of the probe and diameter, there was no significant difference between the two depths in phantom subjects with two kinds of stiffness (stiffness 1 *p* = 0.261, stiffness 2 *p* = 0.950) (Figure 5).

### Accuracy of the shear wave elastography measurements

The CVs show that the SWE measurement of four probes have a good reproducibility (Table 1). For stiffness 1: L11-3 0.52–2.92%, L14-5 0.3–1.72%, SL15-4 0.27–2.82%, and SL10-2 0.57–2.96%. For stiffness 2: L11-3 0.14–1.86%, L14-5 0.58–7.16%, SL15-4 1.53–3.06%, and SL10-2 1.34–3.02%.

The measurements on both probes were lower than the theoretical values of two kinds of stiffness (Figure 3). For the phantom with stiffness 1, the percentage error ranges of L11-3, L14-5, SL15-4, and SL10-2 are 19.13–28.14%, 14.72–27.89%, 0.73–12.23%, and 12.91–25.13%, respectively. For the phantom with stiffness 2, the percentage error ranges of L11-3, L14-5, SL15-4, and SL10-2 are 24.45–31.49%, 27.78–40.11%, 3.80–22.13%, and 12.67–25.41%, respectively.

## Discussion

In this study, we found that SWE measurements can be significantly different according to the machine, probe, and diameter. While the depth has no significant effect on SWE within superficial depth.

The Quantitative Imaging Biomarkers Alliance (QIBA) of the Radiological Society of North America (RSNA) concluded that there were significant differences in SWE measurements between four machines which including FibroScan, Philips, ACUSON S2000, and Aixplorer (25). However, the study did not analyze the effect of the probe on SWE. Another study (20) used aiexplorer, ACUSON S3000, and EPIQ 5 to demonstrate that the probes and machines from different vendors can affect the SWE measurement. To expand the scope of research, our study used two machines from different vendors (Aixplorer and Resona 7), and each machine selected two types of high-frequency linear probes. Different from previous studies, our study focused on E_mean_, i.e., Young’s modulus. Our results show that the difference in the SWE measurement values between the two machines is significant under some conditions. And with the increase in the stiffness of the phantom, the difference between machines becomes significant. In this study, we found that the measurement values between the two probes under the same machine have good consistency, and the difference is not statistically significant. Therefore, we think that we should keep the consistency of the machine when follow-up lesions. In addition, the influence of different vendors should be considered when analyzing the change in elasticity value of lesions. Our results showed that the SWE measurements with all probes were lower than the theoretical values of the phantom which is in agreement with some previous studies (8). We think that the reasons for this phenomenon were as follows: First, the theoretical values of the phantom in this study were measured by Verasonics, which is different from the current commercial ultrasonic machine used in the calculation method of SWV post-processing, which may have led to differences between the two measurements. Second, the stiffness of the matrix around the phantom tumors was very low, and the ratio of the stiffness of the phantom tumors to the matrix was large. After post-processing, the difference between them decreased, which may have also resulted in lower measurements than the theoretical values of the phantom.

To our knowledge, few studies have assessed the effect of the diameter of the subject on SWE. Studies (26) have compared strain elastography and SWE, and they found that target diameter affected all methods, and the largest effect was seen in SWV measurements. Our results show that the measurement values of the phantom subjects with 15 mm diameter are higher than that of 10 and 18 mm and are closer to the theoretical values. Some studies (27) discussed the influence of the “size effect” on SWE. The study shows that when the dimension of the lesion is smaller than 15 mm, the SWE measurement will be affected by the “size effect.” Since Young’s modulus is derived from the formula *E* = 3ρ.SWV^2^ (28, 29), the measured tissue size must be much larger than the shear wavelength (27, 28). The elasticity values of the lesions will be heavily underestimated when the size of the lesion is too small. However, our results show that the measurement value of 18 mm phantom is also significantly smaller than the theoretical value. After analyzing the SWE image, we found that while measuring the 18 mm phantom, the ROI contained the matrix around the phantom tumors, which led to a low measurement value. With an increase in the diameter, it is difficult to create a sphere in a strict sense, and the bottom of the sphere will be flat. In this condition, if the diameter of the ROI is selected according to the diameter of the phantom, the matrix will inevitably be included. In addition, our study also found that the difference between the 10 and 15 mm increased as the phantom become harder. Zhang et al.’s study (5) is consistent with our study. Carlsen et al. (21) found that SWV diminished with decreasing target diameter for 80- and 45-kPa subjects, whereas the SWV increased for 14- and 8-kPa subjects. The above results show that there is an interaction between diameter and stiffness, and the effect of diameter on SWE was related to the stiffness of the tissue.

With respect to the effect of depth on SWE, previous studies (20, 21, 29, 30) have suggested that the SWV could decrease with an increase in the acquisition depth. However, our study found that the SWE measurements did not differ significantly between the depths of 15 and 25 mm. We think that this is because the depth range set in previous studies is larger than our study, and in the superficial depth range of 15 and 25 mm, shear wave elasticity does not decay significantly. Previous studies (8, 25, 31) have shown that the reproducibility of SWE measurement decreases when depth increased. Therefore, we should consider the influence of depth on measurement accuracy when lesions are located deep. Some studies (8, 20, 24, 31, 32) have suggested that when the depth of the lesion is more than 4–5 cm, the linear array probe should not be used for elasticity measurement.

The study has several limitations. We used the phantom for experiments; however, the human tissue is more complex, and patient age, fat content, respiration, and other factors can influence the measurements. Therefore, this experiment cannot mimic clinical cases. In addition, the purpose of this study was to discuss the influence of factors, such as diameter, depth, and stiffness, on SWE under superficial conditions, Therefore, the depth ranges chosen were not wide enough. Furthermore, stiffnesses 1 and 2 were medium, and SWE under other softer or harder conditions was not explored. Finally, the sample size of this experiment was relatively inadequate; thus, to obtain more reliable results, a number of tests should be conducted in future experiments.

## Conclusion

This study demonstrated that the SWE measurements could have a good consistency when the probes were under the same machine. Caution should be used when the diameter of the lesion is smaller than 15 mm and the degree of influence is related to the stiffness. Finally, the SWE is affected by depth and can be ignored when the lesion is located superficially.

## Data availability statement

The original contributions presented in this study are included in the article/supplementary material, further inquiries can be directed to the corresponding author.

## Author contributions

QC wrote the first draft of the manuscript. BS collected and collated the data. YZ made the phantoms. XH edited and revised the manuscript. All authors reviewed the manuscript and approved the submitted version.
